# Both Moderate and Heavy Alcohol Use Amplify the Adverse Cardiovascular Effects of Smoking in Young Patients with Hypertension

**DOI:** 10.3390/jcm12082792

**Published:** 2023-04-09

**Authors:** Paolo Palatini, Lucio Mos, Francesca Saladini, Olga Vriz, Claudio Fania, Andrea Ermolao, Francesca Battista, Mattia Canevari, Marcello Rattazzi

**Affiliations:** 1 Department of Medicine, University of Padova, 35128 Padova, Italyfrancesca.battista@unipd.it (F.B.);; 2 San Antonio Hospital, 33038 San Daniele del Friuli, Italy; luciomos@libero.it (L.M.); olgavriz@yahoo.com (O.V.);; 3 Cittadella Town Hospital, 35013 Cittadella, Italy; saladinifrancesca@gmail.com; 4 Villa Maria Hospital, 35138 Padova, Italy; faniaclaudio@gmail.com

**Keywords:** alcohol, smoking, cardiovascular, risk, hypertension

## Abstract

Aim: To evaluate the association of alcohol and smoking combined with cardiovascular and renal events and investigate whether moderate and heavy alcohol consumption have a different impact on this association. Methods: The study was conducted in 1208 young-to-middle-age stage 1 hypertensive patients. Subjects were classified into three categories of cigarette smoking and alcohol use, and the risk of adverse outcomes was assessed over a 17.4-year follow-up. Results: In multivariable Cox models, smoking showed a different prognostic impact on alcohol drinkers and abstainers. In the former, an increase in the risk of cardiovascular and renal events was observed compared to nonsmokers (hazard ratio, 2.6, 95% CI, 1.5–4.3, *p* < 0.001), whereas in the latter, the risk did not achieve the level of statistical significance (*p* = 0.27) with a significant interaction between smoking and alcohol use (*p* < 0.001). Among the heavy smokers who also drank alcoholic beverages, the hazard ratio from the fully adjusted model was 4.3 (95% CI, 2.3–8.0, *p* < 0.0001). In the subjects with moderate alcohol consumption, the risk of smoking and alcohol combined was similar to that found in the whole population (hazard ratio, 2.7; 95% CI, 1.5–3.9, *p* < 0.001). Among the subjects with heavy alcohol consumption, the hazard ratio was 3.4 (95% CI, 1.3–8.6, *p* = 0.011). Conclusion: These findings indicate that the detrimental cardiovascular effects of smoking can be worsened by concomitant alcohol use. This synergistic effect occurs not only for heavy alcohol consumption but also for moderate use. Smokers should be aware of the increased risk associated with concomitant alcohol consumption.

## 1. Introduction

The unhealthy effects of smoking, including an increased risk for several types of cancer, chronic obstructive pulmonary disease, coronary heart disease, and cardiovascular disease in general, have been well documented since the publication of the seminal Framingham studies [1,2,3,4]. Tobacco use remains the leading cause of morbidity and mortality in the United States [5] and is estimated to cause approximately 8 million deaths per year throughout the world [6]. Conversely, quitting smoking is associated with a significant reduction in the risk of coronary heart disease and stroke [7].

Alcohol use has also been found to increase the risk of developing serious diseases, including cancer, liver cirrhosis, hypertension, and hemorrhagic stroke [8,9]. However, the relationship between alcohol intake and cardiovascular disease is controversial because the results of the literature have been inconsistent. Although epidemiological studies have shown that excessive drinking is harmful to humans, evidence shows that mild-to-moderate alcohol consumption may even reduce cardiovascular risk [10,11,12,13,14]. Indeed, meta-analysis studies have advocated the health benefit of light to moderate alcohol consumption related to cardiovascular disease [15,16].

Epidemiological data suggest that smokers are at a higher risk of greater alcohol use patterns, including greater consumption and higher binge-like alcohol use [17,18]. Indeed, daily smokers have been shown to be more likely to meet the criteria for hazardous drinking and other alcohol-related diagnoses [19,20]. A body of evidence indicates that the detrimental effects seen with the singular abuse of smoking or alcohol use also increase substantially as a result of their co-use [21]. A strong synergistic effect between smoking and alcohol has been found in the induction of several types of cancer [22,23]. The ominous multiplicative consequences of concurrent use extend to other clinical conditions, such as liver cirrhosis, pancreatitis, and psychiatric co-morbidity [24,25,26]. Recent research has also documented a significant increase in hypertension risk due to tobacco–alcohol interaction [27,28].

However, little is known about the combined effect of alcohol and smoking on cardiovascular events, especially in young individuals, despite emerging concerns about the cardiovascular health of this segment of the population. Recent data indicate that treatment and control of major risk factors for cardiovascular disease among young adults are far from optimal and suggest that cardiovascular disease may now be increasing in this population [29,30,31].

In a previous analysis of the HARVEST population, we observed an interactive effect of alcohol and smoking on the risk of cardiovascular events in 18- to 45-year-old individuals [32]. The aim of the present analysis was to evaluate the association of alcohol and smoking combined with cardiovascular and renal events over a longer follow-up and investigate whether moderate and heavy alcohol consumption have a different impact on this association.

## 2. Methods

### 2.1. Subjects

The HARVEST is a multicenter prospective observational study conducted in 17 hypertension units in Italy that began on 1 April 1990 [33,34,35]. The study participants are 18- to 45-year-old never-treated patients screened for stage 1 hypertension (systolic blood pressure (BP) ≥ 140 mmHg and/or diastolic BP ≥ 90 mmHg). Subjects with diabetes, nephropathy, cardiovascular disease, neoplastic diseases, and any other serious clinical condition were excluded [33,34,35]. Consecutive patients with the above-mentioned clinical characteristics were eligible for recruitment and were sent to the referral centers by their general practitioners. Participants were recruited for five years after the beginning of the study. A total of 1208 participants who had at least 6 months of follow-up were included in this analysis. Patient data and blood and urine samples were periodically sent to the coordinating center in Padova, Italy, where they were processed.

### 2.2. Data Collection

The questionnaire captured data pertaining to the participants’ demographics, personal and family health, medical history, and lifestyle habits, which included information about smoking, alcohol intake, coffee consumption, and physical activity. Participants were classified into three categories according to the daily number of cigarettes smoked: nonsmokers, 1–10 cigarettes/day, and >10 cigarettes/day. They were also divided into three categories of alcohol use: 0 g/day, <50 g/day, and ≥50 g/day. Coffee consumption was defined according to the number of caffeine-containing coffees drunk per day. For the present analysis, participants were divided into two categories: coffee drinkers and nondrinkers. Information on physical activity was collected using a previously published classification [33,34]. A family history of cardiovascular disease was defined as stroke, myocardial infarction, or sudden death before the age of 60 in a first-degree relative. More details about the interview and lifestyle assessment have been reported elsewhere [32,33,34,35]. All subjects underwent physical examination, anthropometry, blood chemistry, and urine analysis. Body mass index (BMI) was considered as an index of adiposity (weight divided by height squared). BP was measured in the office using the auscultatory method, and the mean of six readings obtained during two visits performed 2 weeks apart was considered as the baseline office BP. Twenty-four-hour ambulatory BP monitoring was performed using the A&D TM-2420 model 7 (Tokyo, Japan) or the ICR Spacelabs 90207 (Redmond, WA, USA). Measurements were taken every 10 min during the day (06.00–23.00 h) and every 30 min during the night (23.00–06.00 h). The procedures followed were in accordance with institutional guidelines, and the study was approved by the Ethics Committee of the HARVEST and by the Department of Clinical and Experimental Medicine of the University of Padova. Written informed consent was given by the participants.

### 2.3. Follow-up and Outcomes

During the follow-up, visits were scheduled every 6 months. Antihypertensive treatment was started following the recommendations of international guidelines available at the time of the visit (see Supplementary Materials). Then, treated and untreated subjects continued to be checked at 6-month intervals. For survivors who were lost to follow-up, the date of the last available visit was considered. Survival time was defined as the period from the date of the first visit to the date of first adverse event. Detailed information on the follow-up procedures has been reported elsewhere [32,33,34,35].

Cardiovascular events included fatal and nonfatal strokes, fatal and nonfatal ST-elevated acute myocardial infarction, non ST-elevated acute coronary syndromes, any myocardial revascularization procedure, heart failure needing hospitalization, any aortic or lower limb revascularization procedure, and development of permanent atrial fibrillation. Renal events were defined as chronic kidney disease at stage 3 or higher (estimated glomerular filtration rate < 60 mL/min/1.73 m^2^). We ascertained vital status and the incidence of fatal and nonfatal events from medical records and interviews with attending physicians and patients’ families.

### 2.4. Data Analysis

Quantitative variables were reported as mean ± SD unless specified. For follow-up duration, the median and interquartile range (IQR) were calculated. Categorical variables were reported as percentages and differences in the distribution were tested by χ^2^ test. Differences in the distribution of continuous variables across groups were tested by ANCOVA test adjusting for age and sex. Lifestyle factors were modeled as time-dependent categorical variables in Cox proportional hazards regressions adjusting for age and sex. Subsequently, coffee consumption (yes/no), physical activity (yes/no), parental history of cardiovascular disease, BMI, total cholesterol, average 24 h systolic and diastolic BPs, and incident hypertension needing antihypertensive treatment diagnosed during the follow-up were also included. No violations to the proportional hazards assumption were detected by inspection of survival curves. Hazard ratios and corresponding two-sided 95% confidence intervals were derived from the regression coefficients in the Cox models. Tests for interaction effects were conducted in multivariate regression analyses by adding the product of smoking and alcohol in the models. A two-tailed probability value ≤0.05 was considered significant. Analyses were performed using Systat version 12 (SPSS Inc., Evanston, IL, USA) and MedCalc version 20.218 (MedCalc Software Ltd., Ostend, Belgium).

## 3. Results

Clinical characteristics of the subjects stratified by smoking and alcohol use are reported in Table 1. Compared with nonsmokers who abstained from alcohol consumption, participants who smoke and drank alcohol were slightly older, were more frequently male, and had higher 24 h systolic BP.

### 3.1. Relationship between Lifestyle Factors

**Figure 1:**
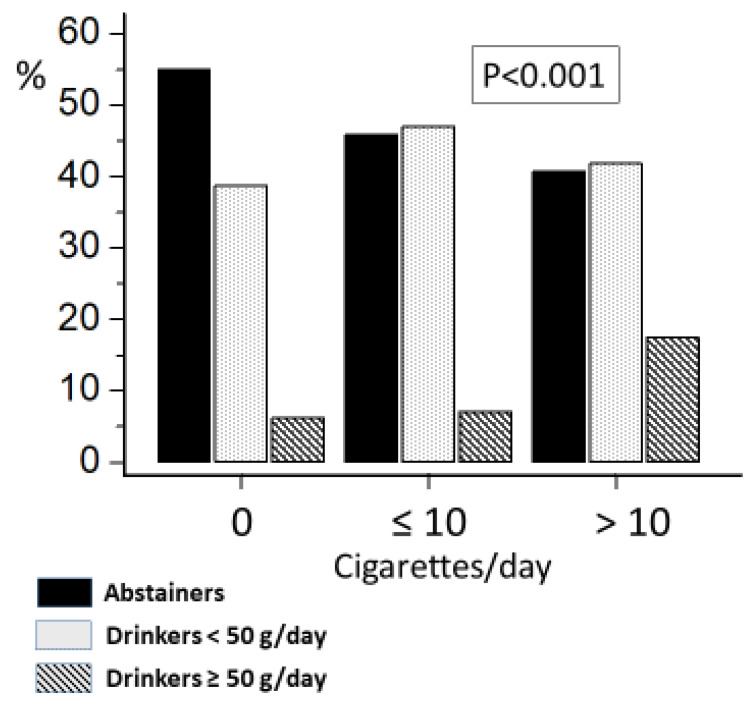
Frequency of alcohol abstainers, moderate alcohol drinkers (<50 g/day), and heavy alcohol drinkers (≥50 g/day) according to smoking category in 1208 participants from the HARVEST study.

**Figure 2:**
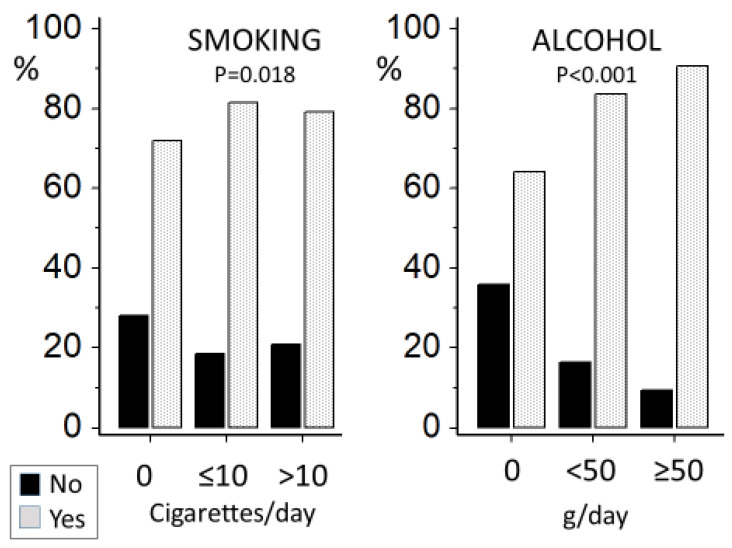
Frequency of coffee users (yes/no) according to alcohol and smoking category in 1208 participants from the HARVEST study.

Smokers were more frequently alcohol drinkers than nonsmokers. Alcohol consumption was proportional to the number of cigarettes smoked per day (Figure 1). Coffee consumption was more frequent in alcohol drinkers than nondrinkers and in smokers than nonsmokers (Figure 2). The highest rate of coffee drinkers was found in the group of smokers who also drank alcohol (Table 1). Nonsmokers who abstained from alcohol were more physically active than the other three groups (Table 1).

### 3.2. Association with Cardiovascular and Renal Events

During a median follow-up of 17.4 (IQR 8.9–23.0) years, there were 108 adverse events (8.9%). Of these, 96 were fatal and nonfatal cardiovascular events (7.9%), and 12 were renal events (1.0%). The rate of total events was higher among the 879 men (N = 88; 10.0%) than the 329 women (N = 20; 6.1%) (chi^2^ 4.5; *p* = 0.033). The most common events were acute coronary syndromes (n = 50; 4.1%), with 4.9% among men and 2.1% among women (chi^2^ 4.6; *p* = 0.032).

**Figure 3:**
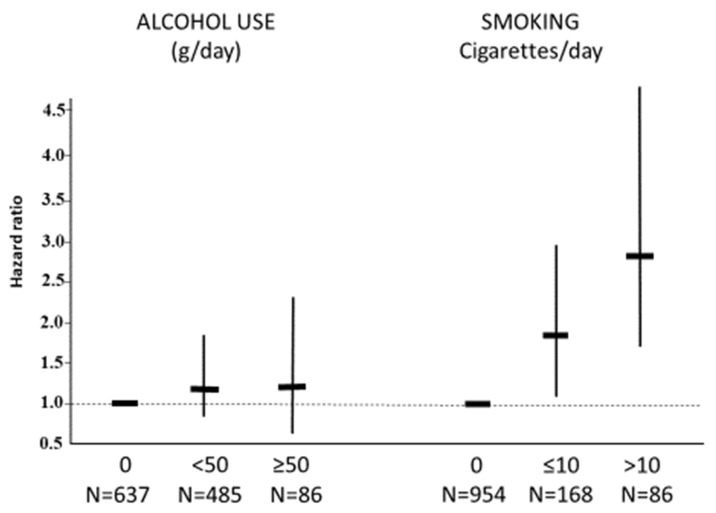
Hazard ratios (95% confidence intervals) for cardiovascular and renal events from multivariable Cox models in the HARVEST participants stratified by tobacco and alcohol categories.

**Figure 4:**
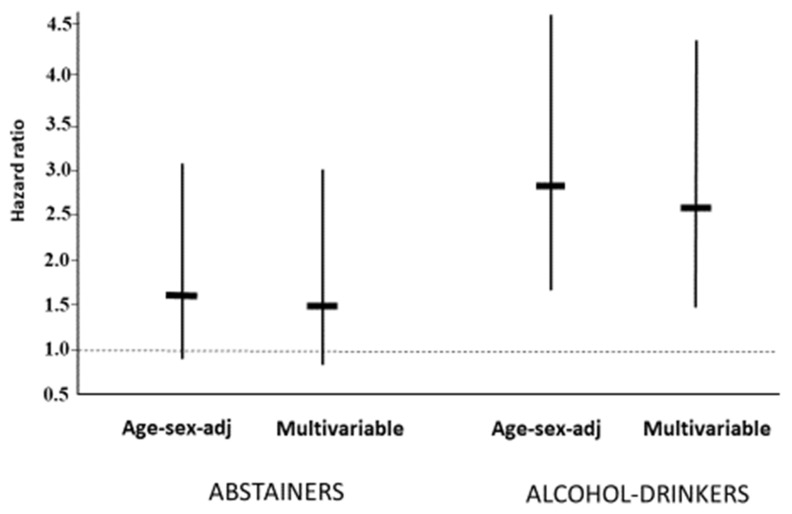
Hazard ratios (95% confidence intervals) for cardiovascular and renal events from multivariable Cox models in the HARVEST participants stratified according to whether they were alcohol drinkers or abstainers. Within each alcohol group, the hazard ratios represent the risk for smokers versus nonsmokers.

The risk of cardiovascular and renal events from a multivariable Cox model including age, gender, BMI, parental history for cardiovascular disease, coffee consumption (yes/no), physical activity (yes/no), total cholesterol, mean 24 h systolic and diastolic BPs, and incident hypertension in the subjects stratified by smoking and alcohol drinking is reported in Figure 3. The risk was slightly higher in alcohol drinkers than abstainers but was not statistically significant. Among the smokers, the risk was increased compared to nonsmokers and was proportional to the number of cigarettes smoked per day (Figure 3). However, smoking showed a different prognostic impact on alcohol drinkers and abstainers. In the former, a clear increase in risk was observed compared to nonsmokers (hazard ratio, 2.6, 95% CI, 1.5–4.3, *p* < 0.001). In the latter, the risk did not achieve the level of statistical significance (hazard ratio, 1.5; 95% CI, 0.7–3.0, *p* = 0.27) with a significant interaction between smoking and alcohol use (*p* < 0.001) (Figure 4). Among the heavy smokers (>10 cigarettes/day) who also drank alcoholic beverages, the hazard ratio from the fully adjusted model was 4.3 (95% CI, 2.3–8.0, *p* < 0.0001).

**Figure 5:**
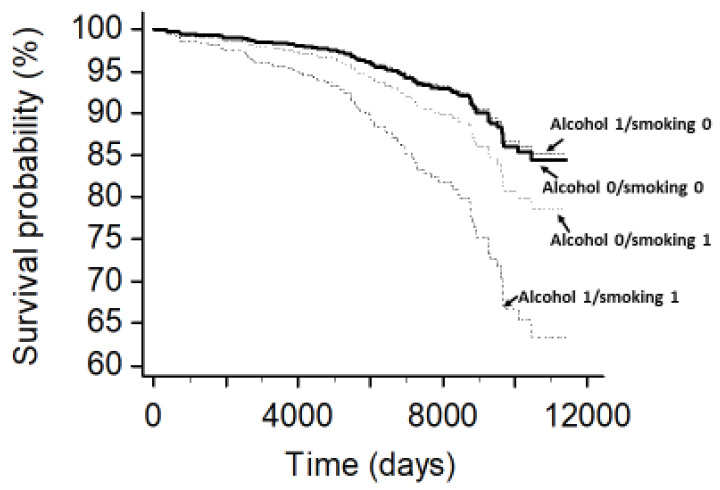
Adjusted survival curves from the Cox multivariable model for the HARVEST participants stratified according to smoking (yes/no) and alcohol use (yes/no). Major adverse cardiovascular and renal events were considered as the outcome variables.

The survival curves for the participants stratified according to smoking and alcohol combined are reported in Figure 5. Taking the group of nonsmokers/nonalcohol drinkers as the reference, a clear increase in the risk of adverse outcomes was observed in smokers who drank alcoholic beverages. No increase in risk was observed for the group of alcohol drinkers who did not smoke. The inclusion of incident hypertension in the survival models did not attenuate the synergistic effect of smoking and alcohol on the risk of events.

### 3.3. Risk in Smokers by Level of Alcohol Consumption

The rate of cardiovascular and renal events was 6.3% in nonsmokers/nonalcohol drinkers, 17.4% in the group of smokers with moderate alcohol consumption (<50 g/day), and 22.2% in the smokers with heavy alcohol consumption (≥50 g/day). In a sensitivity Cox analysis, the risk of smoking and alcohol combined in the subjects with moderate alcohol consumption (N = 1121, number of events = 93) was similar to that found in the whole population (hazard ratio, 2.7; 95% CI, 1.5–3.9, *p* < 0.001) (Supplementary Figure S1). Among the subjects with heavy alcohol consumption (N = 723, number of events = 58), the hazard ratio was 3.4 (95% CI, 1.3–8.6, *p* = 0.011) (Supplementary Figure S2).

## 4. Discussion

In this prospective cohort study of young-to-middle-aged subjects with stage 1 hypertension followed for over 17 years, we observed that the detrimental effects of smoking on cardiovascular health were worsened by concomitant alcohol use confirming our previous findings that smoking and drinking can act synergistically to increase cardiovascular risk [32]. In addition, the present results show that this synergistic effect occurs not only for heavy alcohol consumption but also for moderate use.

Tobacco consumption is widely recognized as an important risk factor for cardiovascular disease and multiple chronic noncommunicable diseases [1,2,3,4,5,6] and is the leading cause of death among middle-aged and older men [36]. Thus, there is a need to implement actionable interventions to reduce smoking-related cardiovascular risk. In addition to promoting smoking cessation, identifying risk factors that amplify the detrimental effect of smoking may be of help to reduce the burden of tobacco use on public health.

According to the American Heart Association, ideal cardiovascular health should include the combination of the 7 factors that compose the so-called “Life’s Simple 7”: not smoking, having a healthy diet pattern, adequate physical activity, healthy body weight, and healthy BP, cholesterol, and blood glucose in the absence of pharmacological treatment [37]. Alcohol was not included in this list because findings reported in the literature were often inconsistent across studies.

A strong synergistic effect between smoking and alcohol has been found in several clinical conditions, including some types of cancer [22,23,24], liver cirrhosis, pancreatitis, and psychiatric co-morbidity [24,25,26]. Two recent studies have documented a significant increase in the risk of hypertension due to the joint effect of tobacco and alcohol [27,28], emphasizing the importance of controlling the concurrence of smoking and alcohol consumption to prevent hypertension. However, little is known about the combined effect of these two risk factors on cardiovascular events. In a cohort of Korean adults aged 20–65 years with elevated BP, smoking and alcohol consumption, independently and jointly, were found to be associated with the risk of cardiovascular disease, but the interactive effect of these two lifestyle factors was not tested [38].

A J-shaped relationship between alcohol use and cardiovascular mortality has been described in several studies and meta-analyses, suggesting an increase in risk among heavy drinkers and a protective effect among light users [9,10,11,12,13]. However, recent studies suggest that the reduction in cardiovascular risk is a result of global lifestyle changes and that any reduction in alcohol consumption is beneficial [39]. In the present study, among nonsmokers, both alcohol categories were not associated with increased cardiovascular risk. In contrast, among the smokers, the multiplicative adverse effect of alcohol was found for both heavy and moderate use with hazard ratios of 3.4 and 2.7, respectively. These data are in keeping with those by Shin et al., who found that the adverse effect of alcohol combined with smoking also occurred for moderate alcohol consumption [38]. In a cohort study by Xu et al., nonsmokers with moderate alcohol consumption showed a reduced risk of cardiovascular events; in contrast, no beneficial effect from alcohol was observed in smokers with moderate alcohol use [40].

Tobacco and alcohol use have reciprocal influences on potentiating cravings and interact with each other leading to more frequent use and higher consumption levels [41]. A recent US study has shown that high-risk lifestyle factors tend to cluster in the population and that the joint risk is much higher than the sum of the individual risks [42], highlighting the importance of paying attention to their unhealthy co-effects. The present study confirms that unhealthy lifestyle factors tend to cluster together, as smokers were more frequent alcohol drinkers and had more sedentary habits.

The mechanisms underlying the interactive cardiovascular effects of smoking and alcohol remain elusive. In a previous study, we observed an interactive effect of these two factors on 24 h catecholamine output, suggesting that increased sympatho-adrenergic activity may be a contributing factor to the synergistic cardiovascular effect of tobacco and alcohol use [32]. In addition, it has been shown that both alcohol consumption and smoking trigger the production of carbon monoxide and phenolic free radicals, which have proven pro-oxidant and pro-inflammatory effects, thereby increasing the likelihood of adverse clinical outcomes [43]. Through chronic inflammation and endothelial damage, both tobacco and alcohol consumption can cause a direct or indirect impairment of vascular elasticity and promote atherosclerosis [44,45].

## 5. Limitations

We acknowledge that the present study has several limitations. First, in our study, the status of lifestyle factors was self-reported by the participants and thus subject to misclassification. However, health behaviors were checked frequently during the follow-up, showing that lifestyle habits were constant and well-reported [32,33]. Second, the evaluation criteria of lifestyle factors in the HARVEST differ from those of other reports, which might cause differences between the studies. This limitation pertains particularly to the definition of heavy and moderate drinkers, which is inconsistent among different studies. Third, we enrolled only Caucasians and were unable to perform subgroup analyses by race or ethnicity. In addition, we could not estimate differences between men and women because of the smaller sample size and the low number of events among the women. Therefore, the present results may not be applicable to women or people of other racial backgrounds.

## 6. Conclusions

The present findings indicate that there is an important synergistic effect between tobacco and alcohol consumption on cardiovascular and renal outcomes in young hypertensive subjects. The multiplicative risk associated with smoking and alcohol drinking also occurred for moderate alcohol consumption, emphasizing the importance of controlling the concurrence of unhealthy lifestyle factors. Strategies for improving lifestyle behaviors are well recognized and should be applied and reinforced, particularly in young patients with hypertension. Indeed, increases in heart disease and cardiovascular mortality has been reported in recent years in younger adults, which reflect recent unfavorable trends in cardiovascular risk determinants and events [29,46]. Tobacco product use remains high among youth in Western countries [47], calling for the implementation of population-based tobacco control strategies in this segment of the population. However, only a minority of smokers state they want to quit at some point in time [48]. Thus, smokers should be aware that quitting alcohol consumption would reduce the detrimental effects of tobacco use.

## Figures and Tables

**Table 1:** Characteristics of the participants grouped according to smoking and alcohol use (yes/no).

Variable	Alcohol No Smoking No (N = 525)		Alcohol Yes Smoking No (N = 429)		Alcohol No Smoking Yes (N = 112)		Alcohol Yes Smoking Yes (N = 142)		
	Mean	SD	Mean	SD	Mean	SD	Mean	SD	*p*-Value
Age, years	30.8	8.8	35.4 *	7.7	32.4	8.0	34.8 *	7.9	<0.001
BMI, kg/m^2^	25.0	3.7	25.6	2.9	25.7	3.4	25.4	3.1	0.07
Office SBP, mmHg	145.9	10.7	145.6	10.0	144.5	10.7	144.5	11.2	0.37
Office DBP, mmHg	92.9	6.1	94.4	5.1	93.3	5.4	93.3	6.1	0.26
Heart rate, bpm	75.7	9.6	73.4	9.1	75.3	9.6	73.1	9.7	0.13
Cholesterol, mg/dL	193.4	36.7	201.2	39.0	197.2	38.2	200.2	39.9	0.92
24-h SBP, mmHg	130.3	10.9	131.2	10.2	131.4	12.5	133.8 †	10.6	0.014
24-h DBP, mmHg	81.7	8.2	81.8	8.0	81.3	8.4	82.3	8.1	0.60
Sex, male %	64.8	--	81.2	--	60.7	--	85.2	--	<0.001
Coffee use, yes %	62.7	--	83.5	--	70.5	--	88.7	--	<0.001
Physical activity, yes %	43.0	--	35.7	--	29.5	--	39.4	--	0.02
MACE, yes %	6.3	--	8.6	--	9.8	--	18.3	--	<0.001

* *p* < 0.001 versus alcohol no/smoking no and † *p* < 0.01 versus alcohol no/smoking no, according to a Bonferroni-corrected post hoc test. MACE indicates major adverse cardiovascular and renal events.

## Data Availability

The data that support the findings of this study are available on reasonable request from the HARVEST study group.

## Supplementary Materials

The following supporting information can be downloaded at: https://www.mdpi.com/article/10.3390/jcm12082792/s1, Figure S1: Adjusted survival curves from the Cox multivariable model for the HARVEST participants stratified according to smoking (yes/no) and moderate alcohol use (yes/no). Major adverse cardiovascular and renal events were considered as the outcome variable; Figure S2: Adjusted survival curves from the Cox multivariable model for the HARVEST participants stratified according to smoking (yes/no) and heavy alcohol use (yes/no). Major adverse cardiovascular and renal events were considered as the outcome variable. References [49,50,51,52] are cited in the supplementary materials.
