# Supplementary material for: Both Moderate and Heavy Alcohol Use Amplify the Adverse Cardiovascular Effects of Smoking in Young Patients with Hypertension
Source: J Clin Med. 2023 Apr 9;12(8):2792. doi: 10.3390/jcm12082792 (PMC10142645; doi:10.3390/jcm12082792)
Supplement: Supplementary file 1 [file jcm-12-02792-s001.zip › jcm-2320363-supplementary.pdf]

Methods: Identification of the patients who needed antihypertensive treatment.

In the HARVEST study, the operational threshold level for identifying participants who needed antihypertensive treatment changed over time in keeping with the guidelines available at the time of patient assessment. In 1990, when we started the study, the British Hypertension Society stipulated that eligibility for antihypertensive medication was progression to grade II hypertension (supine office systolic blood pressure ≥160 mm Hg and/or supine office diastolic blood pressure ≥100 mm Hg) during the first year of follow-up (1,2). Later on, the 1999 ISH/WHO guidelines for patients at low cardiovascular risk, such as the participants in the present study, established that treatment should be given to subjects with a supine office systolic blood pressure ≥150 mm Hg and/or supine office diastolic blood pressure ≥95 mm Hg in two consecutive visits (3). After the publication of the 2003 European Society of Hypertension–European Society of Cardiology guidelines for the management of arterial hypertension (4), which adopted the 140/90 mmHg cut-off also for subjects at low risk, we finally used the 140/90 mmHg threshold.

References

1. Treating mild hypertension. Report of the British Hypertension Society working party. BMJ. 1989;298:694-8.
2. Sever P, Beevers G, Bulpitt C, Lever A, Ramsay L, Reid J, et al. Management guidelines in essential hypertension: report of the second working party of the British Hypertension Society. BMJ. 1993;306:983-7.
3. 1999 World Health Organization-International Society of Hypertension Guidelines for the Management of Hypertension. Guidelines Sub-Committee. Blood Press Suppl. 1999;1:9-43.
4. 2003 European Society of Hypertension-European Society of Cardiology guidelines for the management of arterial hypertension. European Society of Hypertension-European Society of Cardiology Guidelines Committee. J Hypertens. 2003;21:1011-1053.

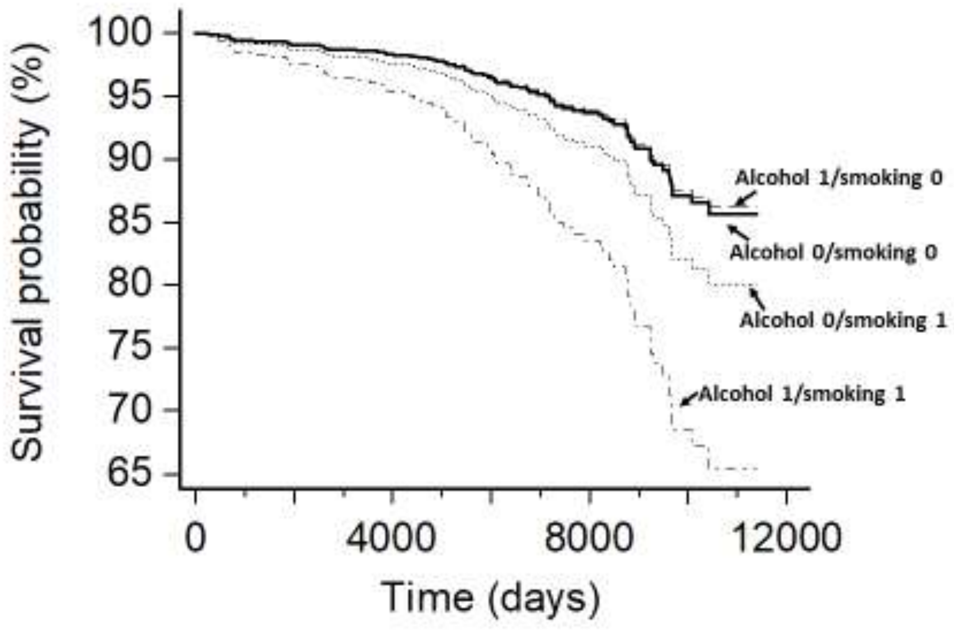

Supplementary Figure S1. Adjusted survival curves from the Cox multivariable model for the HARVEST participants stratified according to smoking (yes/no) and moderate alcohol use (yes/no). Major adverse cardiovascular and renal events were considered as the outcome variables.

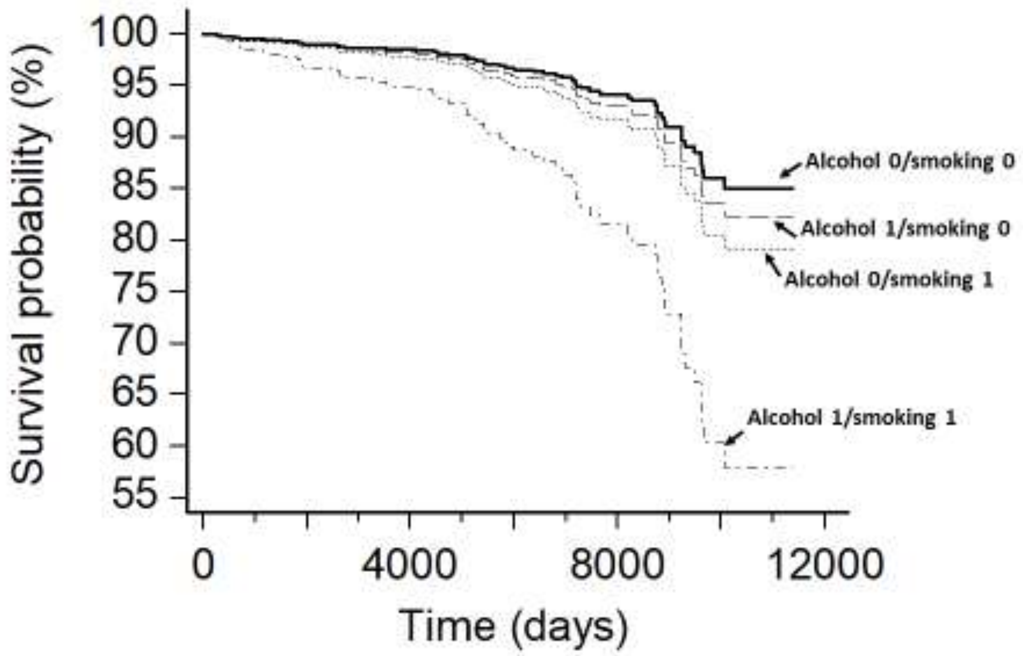

Supplementary Figure S2. Adjusted survival curves from the Cox multivariable model for the HARVEST participants stratified according to smoking (yes/no) and heavy alcohol use (yes/no). Major adverse cardiovascular and renal events were considered as the outcome variables.
